# A comparison of lower extremity squat, lunge, and hip hinge kinematics between marker based and markerless motion capture systems

**DOI:** 10.1177/17543371251348697

**Published:** 2025-07-02

**Authors:** Kristie Liu, Steven Hirsch, Pratham Singh, Timothy CY Chan, Timothy A Burkhart, Michael G Hutchison

**Affiliations:** 1 Faculty of Kinesiology and Physical Education, University of Toronto, Toronto, ON, Canada; 2 Department of Mechanical and Industrial Engineering, University of Toronto, Toronto, ON, Canada

**Keywords:** Markerless motion capture, kinematics, computer vision, movement measurement, pose estimation

## Abstract

The resource-intensive nature of traditional marker-based motion capture systems limits opportunities for quantitative motion analysis. However, the advancement of markerless motion capture technology yields tremendous promise for accessible kinematic analyses beyond conventional research settings. This work compared the lower limb kinematics measured by a neural network-driven markerless motion capture system to those from a standard marker-based motion capture system during squat, hip hinge, and reverse lunge tasks. Fourteen adults performed three repetitions of each movement while being recorded simultaneously by two iPads and 17 infrared optical motion capture cameras. The mean peak cross-correlation values indicated high agreement for knee and hip flexion (>0.95) and poor agreement for hip adduction, knee varus, and internal rotation (<0.49) for all tasks; agreement for hip internal rotation and ankle flexion was task dependent (0.27–0.97). The average root mean square error indicated joint-specific offsets between systems, as values ranged from 6.43° to 12.32° for the knee, 11.25° to 17.35° for the hip, and 21.51° to 25.67° for the ankle. These findings suggest that, while the markerless system demonstrates the ability to capture gross motor patterns in the sagittal plane, further refinement of the underlying models is needed to improve the validity of the system’s measurements.

## Introduction

Human kinematic analysis is the measurement of motion of rigid bodies in the musculoskeletal system and is a key tool for clinical and research biomechanics. Such analyses are important for providing quantitative metrics of movement, in contrast to qualitative visual evaluations. Optical motion capture systems are utilized to estimate the relative positions and orientations of body segments to calculate movement kinematics. Despite the tremendous insights that can be gained from kinematic analysis, the resources required for traditional marker-based motion capture (fixed lab space, expensive specialized cameras, trained personnel, specific software licenses) limit the opportunity for its application outside specialized research laboratories.^1,2^

Most contemporary optical motion capture utilizes a multicamera stereophotogrammetric video system to obtain the three-dimensional coordinates of passive markers placed on anatomical landmarks.^2^ The position and orientation of a body segment are defined by three noncollinear markers in a local coordinate system that moves relative to a global coordinate system.^1,2^ Traditional marker-based motion capture systems track the positions and orientations of physical retroreflective markers placed on the skin over anatomical landmarks.^2^ Specialized cameras are synchronized with an infrared strobe system to capture the circular reflections of the markers.^2^ Despite the ubiquity of marker-based systems in biomechanics research, soft tissue artifact,^3^ and marker misplacement^4^ represent the two most critical sources of measurement error in human movement analysis using this method. These sources of error originate from the use of skin-affixed markers at the interface of the motion capture system and the body under analysis.^3^

The advancement of computational power and artificial intelligence has facilitated the development of markerless motion capture systems. These systems use computer vision algorithms to identify automatically and estimate the positions of joint centers and other anatomical landmarks in each camera frame.^5–7^ Algorithms have been trained on hundreds of thousands of digital images with keypoints labeled by human supervisors.^5,8^ Comparisons of the three-dimensional measurements between markerless motion capture systems and traditional marker-based systems have generally found agreement in the estimation of joint centers,^5,6,9^ and lower limb global segment angles^5^ and joint angles^5,10,11^; however, significant errors from joint center estimation^6^ can have detrimental effects on downstream calculations such as center of mass position and velocity.^12^ Given the lack of ground truth measurements and the sources of error known to affect marker-based motion capture, markerless motion capture is not expected to yield measurements identical to those of marker-based estimates.

The emergence of markerless motion capture is an exciting innovation for motion analysis. Independence from skin-affixed markers circumvents the error introduced by their use, while tremendously reducing participant preparation time. However, multi-camera markerless motion capture systems that have been previously evaluated require technological expertise (i.e., calibration, synchronization, data processing) that limit “in the wild” implementation of such systems.^13^ Alternative approaches utilizing fewer cameras and more ubiquitous devices (i.e., tablets/smartphones) may provide additional opportunities to explore research questions in applied settings where setting up and synchronizing a recommended eight cameras (three minimum) and a dedicated computer for running the camera software may not be feasible. To facilitate movement analysis, out-of-the-box solutions utilizing a two-camera setup for markerless motion capture have been developed (e.g., OpenCap^14^). Before such systems can be deployed, evaluation against the traditional marker-based motion capture for a range of movements is necessary to understand the strengths and limitations of these portable systems. To date, evaluations of markerless motion capture systems have been limited to walking (healthy and pathological gait), running, squatting, and jumping. For use in clinical or sports settings, it is important to examine their validity during standard athletic movement patterns that put individuals through nearly their full range of motion to understand the limitations of these systems better. Additionally, given the different approaches and sources of error, it is necessary to understand whether the differences between systems are affected by the task being performed.

The objectives of this study were to compare the lower limb kinematics of a two-camera markerless motion capture system against those from a traditional marker-based motion capture system during deep squat, hip hinge, and alternating reverse lunge tasks and to evaluate metrics between tasks.

## Methods

### Markerless motion capture

Uplift Labs (Palo Alto, CA, USA) has developed a markerless motion capture system that uses synchronized video data from two iOS devices to perform 3D motion analysis. First, proprietary neural networks estimate the 2D positions of a set of keypoints in each video. Then, these videos are synchronized, and a Direct Linear Transformation algorithm is used to solve the 3D keypoint locations based on the keypoint projections on the image plane of each camera using a proprietary calibration procedure. A proprietary biomechanical model then ingests these 3D keypoints, whereby an inverse kinematics tool fits a rigid model to the estimated keypoint positions. Uplift then exports joint angles and velocities based on the globally optimal pose.^15^

### Participants

Fourteen recreationally active (≥2.5 h of moderate physical activity per week) young adults were recruited to participate in this study (8 males/6 females; mean (SD) age: 22.92 (2.96) years; height: 1.78 (0.097) m; weight: 76.22 (13.05) kg). Exclusion criteria for participants were having (i) concomitant lower limb injuries/surgeries in the past 12 months affecting gait; (ii) injury to the lower extremity and/or back in the last 6 months; (iii) any current discomfort in the lower extremity and/or back; (iv) been diagnosed with any neurological impairment or vestibular disorder that may affect balance; (v) been diagnosed with a concussion within the past 12 months; (vi) or currently taking any medication that may affect balance. Informed consent was obtained in person using a REDCap form. This study was approved by the University of Toronto’s Health Sciences Research Ethics Board (protocol #43034) on June 10, 2022.

### Sample size

Sample size was estimated using G*Power version 3.1.9.7.^16^ In their supplementary material, Uhlrich et al.^14^ reported the mean and standard deviations (SD) of the RMSE for lower limb and trunk joint angles for different movements using different system configurations. For the squat task using a two-camera set-up, the best configuration resulted in mean and SD RMSE values of 4.5° and 1.5°, respectively. The effect size calculated from these values was 2.67. Given three planned post-hoc pairwise comparisons, a Bonferroni adjusted alpha of 1.67% and a power of 80% necessitated a minimum sample size of *N* = 5 in each group for a two-tailed t-test.

### Experimental protocol

The marker-based motion capture system was a 17-camera Qualisys Motion Capture system (Oqus 500 (Qualisys AB, Gothenburg, Sweden)) recording at 100 Hz. For each participant, the same two researchers placed 40 retro-reflective markers on lower body landmarks according to a modified Plug-In Gait model (Figure 1). A static calibration trial for this system was collected with the participant standing in the middle of the capture volume.

**Figure 1.**
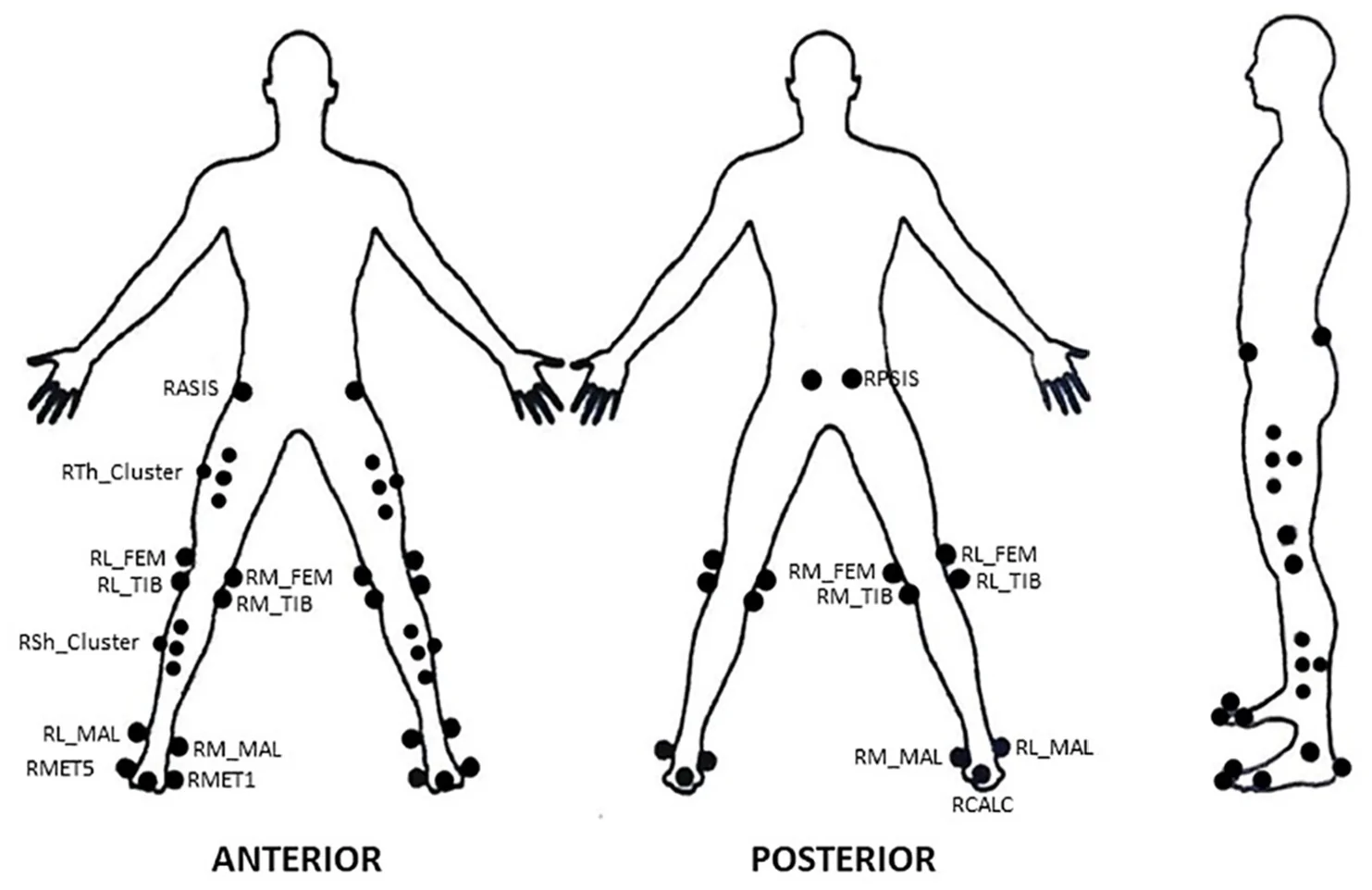
Plug-in gait markerset used for the marker-based motion capture system.

The markerless system synchronized two iPad Pros 6th generation (Apple Inc., Cupertino, California, USA) using peer-to-peer discovery via Wi-Fi connection. The iPads were secured to tripods at a height of 0.9 m and placed 2.5 m anteriorly from the participant at 45° to the left and right of center. This system recorded at 60 Hz and did not require a static trial. The markerset for this system features 23 keypoints (Figure 2), similar to the whole-body annotations used in COCO-WholeBody.^17^ The Uplift system further enhances the model by generating artificial keypoints. The differences between the modeling of the two systems are highlighted in Table 1.

**Figure 2.**
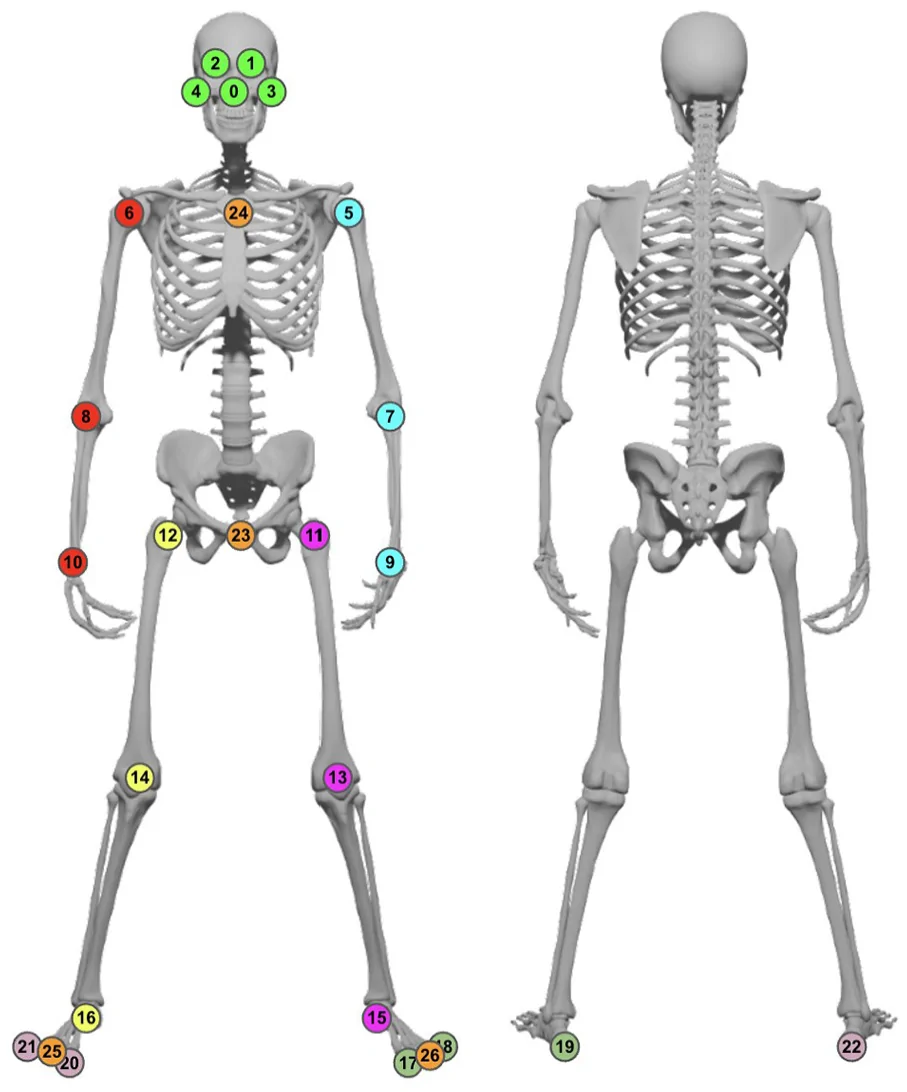
The markerset used for the markerless motion capture system.

**Table 1.** Modeling and signal processing of the markerless motion capture system and marker-based system.

Parameters	Markerless system	Marker-based system
Segments	1 head, 2 upper arms, 2 forearms, 1 trunk, 1 pelvis^a^, 2 thighs, 2 shanks, 2 feet	1 pelvis, 2 thighs, 2 shanks, 2 feet
Joint DoF	3 DoF for neck, shoulders, hips, knee 1 DoF for elbows, knees	6 DoF for hips, knees, ankles
Frame rate	60 Hz	100 Hz
Filtering	Fourth order Butterworth filter	Sixth order Butterworth filter
Modeling approach	Inverse kinematics	6 degrees of freedom

a The model used by the markerless system defines a pelvis segment; however, it lacks the necessary keypoints to model anterior-posterior pelvic tilt. For this reason, hip angle is defined as the thigh segment relative to the trunk in this model.

Participants performed three body weight movements: (1) squat – individuals performed a deep squat, as low as they could comfortably go; (2) hip hinge – with hands by ears and maintaining a flat back, individuals flexed forward at the hips; and (3) alternating reverse lunges – individuals stepped back into a lunge starting with their right leg, returned to the start position, then stepped back with their left leg, before returning to the start position. Each trial consisted of a single repetition (one per leg for the alternating reverse lunge), and three trials were performed per movement.

### Signal processing

Marker-based data were filtered using a zero-lag sixth-order Butterworth filter with a cutoff frequency of 3 Hz, while markerless data were filtered using a zero-lag fourth-order Butterworth filter with a proprietary cutoff frequency; both cutoff frequencies were determined by residual analysis (Winter, 2009). Marker-based data were resampled to 60 Hz using the Fourier method to match the sampling rate of the markerless data. Time series data were synchronized using the offset between the peak left hip flexion values in each trial to align corresponding signals in the trial. Signals were made the same length for each trial by first shifting the start frame of the delayed signal by the length of the offset, then trimming the signals to the length of the shorter signal.

### Data analysis

Post-processing of the tracked marker trajectories from the marker-based motion capture was done in Qualisys Track Manager (v2023.1, Qualisys AB, Gothenburg, Sweden). These data were subsequently exported to Visual3D (C-Motion Inc., Boyds, MD, USA) to calculate joint center positions and lower limb joint angles in the global reference frame. Joint center positions were estimated using the midpoint between the medial and lateral malleoli markers for the ankle, the medial and lateral femoral epicondyles for the knee, and using the Bell and Brand regression equations^18,19^ in Visual3D for the hip.

The Uplift Labs lower body kinematic chain was constrained to have three degrees-of-freedom (DOF) at the hip and knee. Due to the available markers, the output for the ankle angle was limited to plantar-/dorsi-flexion only as a one DOF segment. Additionally, the hip angle was calculated as the orientation of the thigh relative to the trunk. Despite the non-equivalence in hip angle calculations between systems, it was important to compare the Uplift system as-is against a standard markerset for the marker-based system to elucidate the differences between systems.

Joint angles from both systems were calculated according to the sequence of rotations defined by the Grood and Suntay convention, such that the order of rotations follows flexion/extension, abduction/adduction, and internal/external rotation.^20^ The kinematic measurements compared between systems were flexion of the hip, knee, and ankle, hip adduction and knee varus, and hip and knee internal rotation.

All statistical analyses were conducted in Python (v3.9.13) using the scipy (v1.9.1), statsmodel (v0.13.2), and pingouin (v0.5.3) packages. Root mean square error (RMSE) and peak cross-correlation values were calculated for the synchronized signals. Data were then aggregated between the left and right legs for each participant, yielding two data points per trial. The mean and 95% confidence interval of RMSE and peak cross-correlation values were calculated for each movement at each joint. Additionally, two-way repeated measures ANOVAs were used to compare RMSE and peak cross-correlation values of the different tasks; post hoc analyses were conducted using pairwise t-tests with the Bonferroni correction. Bland–Altman plots were used to depict the bias and limits of agreement between the peak joint angle values measured by the two systems. Bias is the mean of the difference between measurements, and the limits of agreement are calculated as bias ±1.96 * standard deviation of the differences. Two-way repeated measures ANOVAs were also used to compare the bias values of the different tasks. Repeated measures correlations^21^ of the peak joint angles were also calculated to provide a measure of the agreement between system measurements.

## Results

### Time series data

The average and 95% confidence interval of RMSE values of time series data for the corresponding lower limb joints of each task across all participants are shown in Table 2. Knee varus had the lowest RMSE values, while the greatest RMSE values occurred in the measurements of ankle flexion.

**Table 2.** Mean (95% CI) of RMSE values for joint angles for each movement task.

Joint	Rotation	Hinge [°]	Lunge [°]	Squat [°]
Hip	Flexion^a^	15.14 [14.15, 16.13]	13.74 [12.49, 14.98]	17.35 [16.11, 18.60]
	Adduction^a^	10.82 [9.54, 12.11]^b^	11.46 [10.26, 12.66]^c^	14.27 [12.76, 15.79] ^b,c^
	Internal rotation^a^	11.25 [10.37, 12. 14]^b^	12.47 [11.88, 13.06]^c^	14.82 [13.90, 15.74] ^b,c^
Knee	Flexion	7.68 [7.09, 8.28]	10.72 [9.80, 11.64]	8.52 [7.35, 9.69]
	Varus^a^	6.43 [5.81, 7.05]^b,c^	8.11 [7.51, 8.71]^b^	8.74 [7.84, 9.63]^c^
	Internal rotation^a^	10.35 [9.28, 11.42]^b^	10.99 [10.11, 11.87]	12.32 [11.25, 13.40]^b^
Ankle	Flexion^a^	25.67 [24.62, 26.73]^b,c^	22.21 [21.11, 23.31]^b^	21.51 [20.28, 22.73]^c^

a Repeated measures ANOVA significantly different.

b and ^c^ used to denote significant pairwise post hoc comparisons between tasks.

Repeated measures ANOVAs were used to compare RMSE values between movement tasks at each joint angle. RMSE values were not significantly different between tasks for knee flexion (*p* = 0.13). Significant differences were found between movement tasks for hip flexion (*p* = 0.03), hip adduction (*p* < 0.001), hip internal rotation (*p* < 0.001), knee varus (*p* = 0.009), knee internal rotation (*p* = 0.04), and ankle flexion (*p* < 0.001); pairwise post hoc comparisons using the Bonferroni correction were conducted between tasks for these joint angles. For hip flexion, post hoc comparisons did not reveal pairwise differences between tasks. RMSE values were significantly different between the hinge and squat (*p* < 0.001) and lunge and squat tasks (*p* = 0.004) for hip adduction, with the greatest RMSE values observed in the squat task. Similarly, post hoc analyses of hip internal rotation revealed pairwise differences between the hinge and squat (*p* = 0.001) and lunge and squat tasks (*p* = 0.04), with the greatest RMSE values occurring in the squat task. Knee varus was significantly different between the hinge and lunge (*p* = 0.02), and hinge and squat tasks (*p* = 0.02), demonstrating greater RMSE values in the squat and lunge tasks relative to the hinge. For knee internal rotation, the hinge and squat tasks were significantly different (*p* = 0.009), with RMSE values of the squat task being greater than those of the hinge. Post hoc pairwise differences of ankle flexion were found between the hinge and lunge (*p* < 0.001), and hinge and squat tasks (*p* < 0.001), with the hinge task having greater RMSE values than the squat and lunge.

Peak cross-correlation values, presented in Table 3, represent the greatest similarity between two signals when one signal is optimally time-shifted relative to the other. Knee and hip flexion demonstrated the highest cross-correlation values across all movements. The peak cross-correlation values for hip internal rotation and ankle flexion were dependent on the task being performed, with the greatest similarity occurring during the squat task for both rotations, and the least similarity occurring during the lunge for hip internal rotation and the hinge for ankle flexion. Cross-correlation values for hip adduction, knee varus, and knee internal rotation were generally poor across all tasks.

**Table 3.** Mean (95% CI) of peak cross-correlation values for joint angles for each movement task.

Joint	Rotation	Hinge	Lunge	Squat
Hip	Flexion	0.99 [0.98, 0.99]	0.98 [0.98, 0.99]	0.98 [0.97, 0.99]
	Adduction	0.49 [0.44, 0.54]	0.40 [0.37, 0.44]	0.48 [0.44, 0.52]
	Internal rotation	0.46 [0.41, 0.50]	0.27 [0.24, 0.30]	0.71 [0.67, 0.75]
Knee	Flexion	0.95 [0.95, 0.96]	0.95 [0.94, 0.96]	0.99 [0.98, 0.99]
	Varus	0.37 [0.34, 0.41]	0.38 [0.34, 0.41]	0.43 [0.38, 0.48]
	Internal rotation	0.41 [0.37, 0.44]	0.34 [0.31, 0.38]	0.47 [0.43, 0.51]
Ankle	Flexion	0.45 [0.41, 0.50]	0.73 [0.69, 0.77]	0.97 [0.96, 0.98]

Repeated measures ANOVAs were used to compare the peak cross-correlation values between tasks at each joint angle. Peak cross correlation values were not significantly different between tasks for hip flexion (*p* = 0.30), hip adduction (*p* = 0.10), and knee varus (*p* = 0.25). Significant differences were found between movement tasks for hip internal rotation (*p* < 0.001), knee flexion (*p* = 0.007), knee internal rotation (*p* = 0.01), and ankle flexion (*p* < 0.001); pairwise post hoc analyses between movement tasks were conducted for these joint angles. For hip internal rotation, significant differences were revealed between the hinge and lunge tasks (*p* < 0.001) and lunge and squat (*p* < 0.001), with the squat task having the greatest peak cross correlation values and lunge having the lowest among the three tasks. For knee flexion, pairwise differences were found between the squat and the hinge (*p* = 0.02) and the squat and the lunge (*p* = 0.03), with the greatest values observed for the squat task. Knee internal rotation for the lunge and squat tasks were significantly different (*p* = 0.01), with the squat having greater peak cross correlation values than the lunge task. Post hoc analyses of ankle flexion revealed pairwise differences between all three tasks (*p* < 0.001); the greatest peak cross correlation values were found in the squat, followed by the lunge, then the hinge.

### Peak joint angles

Bland-Altman plots were created for the peak values measured by each system in each trial (Figures 3–5). Differences between systems were calculated by subtracting the marker-based measurement from the markerless measurement. As such, positive difference values indicate an overestimation of the markerless system relative to the marker-based system. Bias values were predominantly positive, with the only negative bias values observed in hip internal rotation and knee varus for the lunge and squat, and hip adduction and ankle flexion during the hinge and lunge. Bias values ranged from 0.30° (lunge hip flexion) to 20.23° (squat hip flexion). Limits of agreement ranged from 16.69° (squat knee flexion) to 70.07° (squat hip flexion).

**Figure 3.**
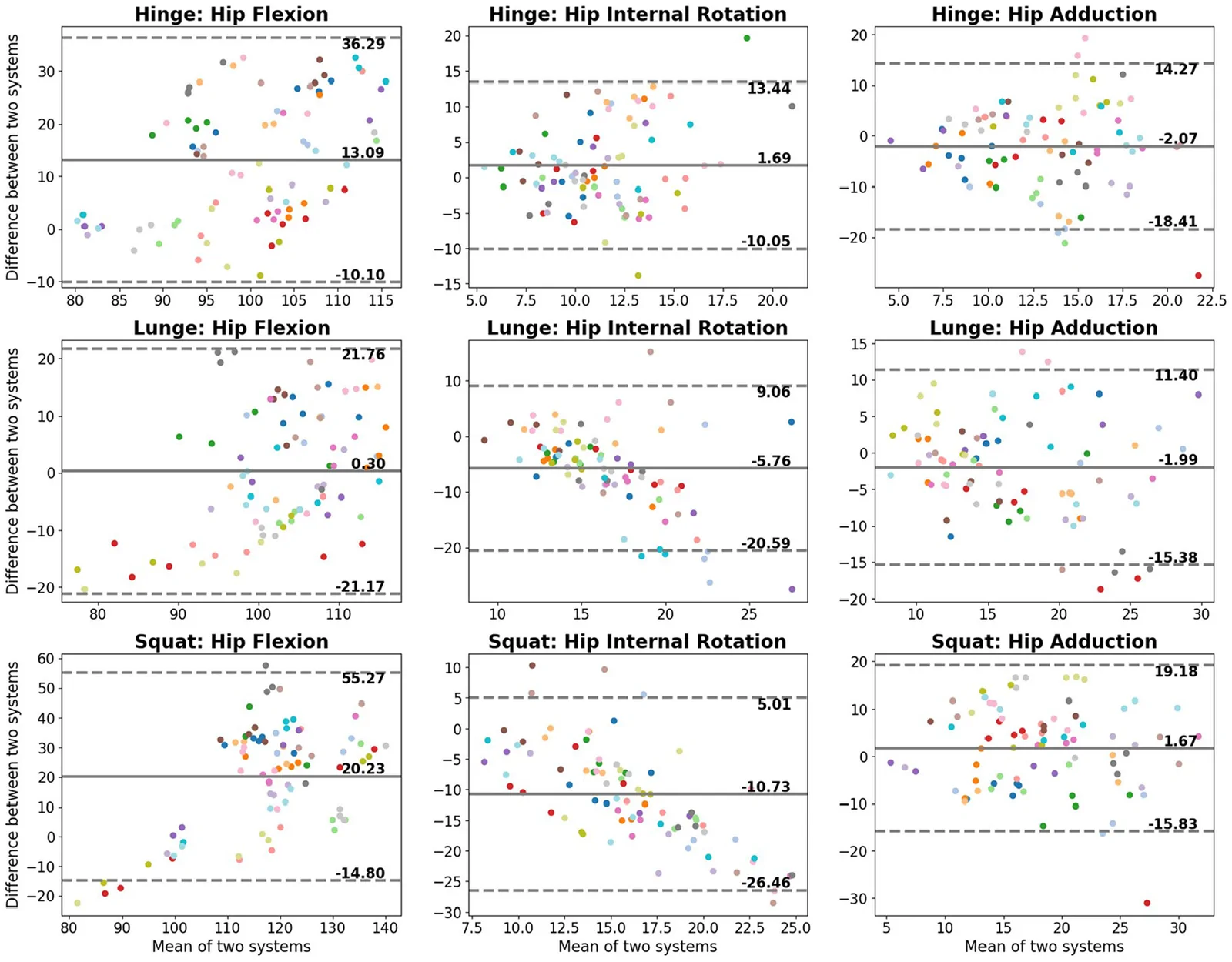
Bland–Altman plots of the peak hip angle values measured by the markerless and marker-based motion capture systems. Differences were calculated as markerless measure − marker-based measure. Bias is the average of the differences between the two systems, and the limits of agreement are ±1.96 standard deviations of the differences.

**Figure 4.**
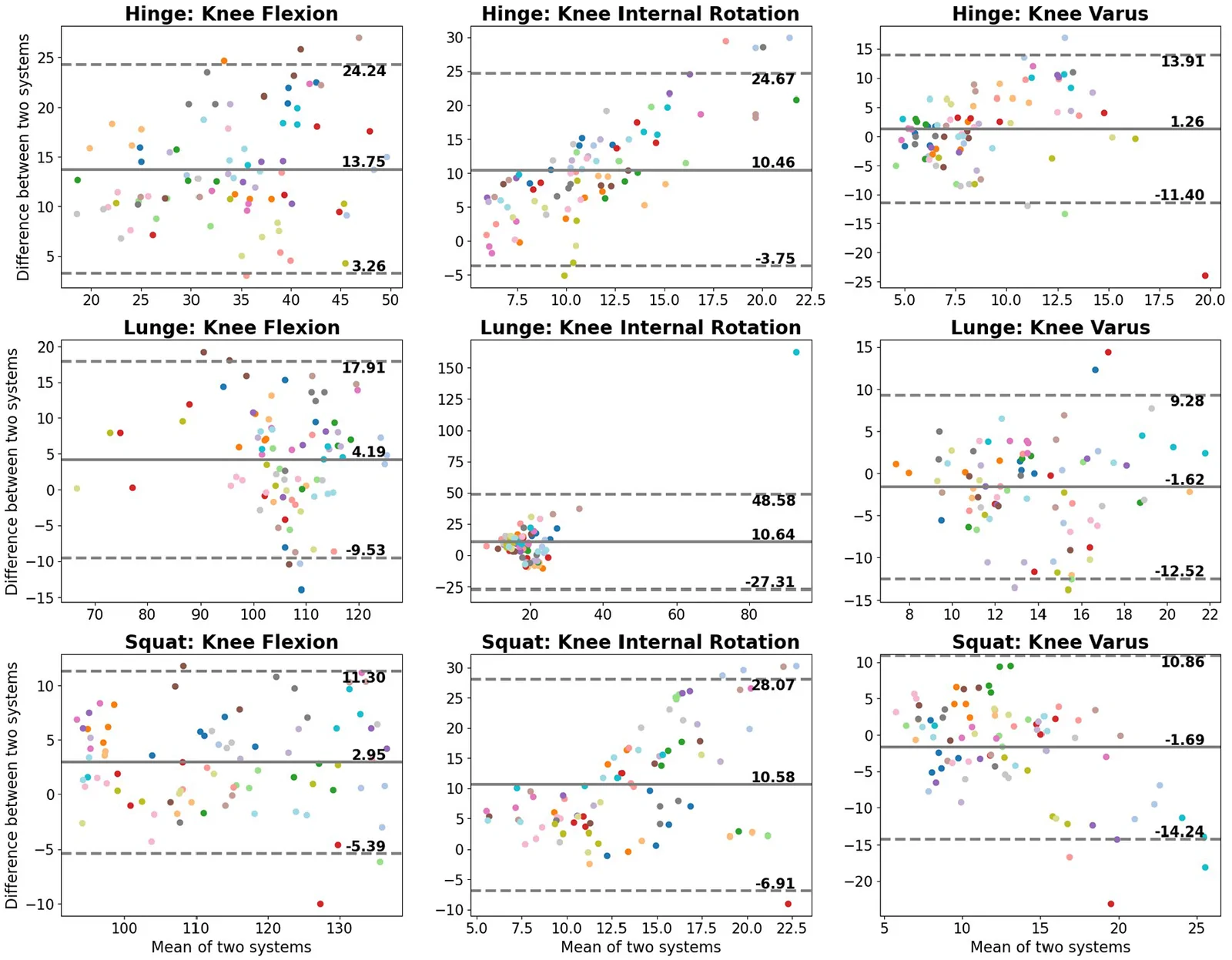
Bland–Altman plots of the peak knee angle values measured by the markerless and marker-based motion capture systems. Differences were calculated as markerless measure − marker-based measure. Bias is the average of the differences between the two systems, and the limits of agreement are bias ±1.96 standard deviations of the differences.

**Figure 5.**
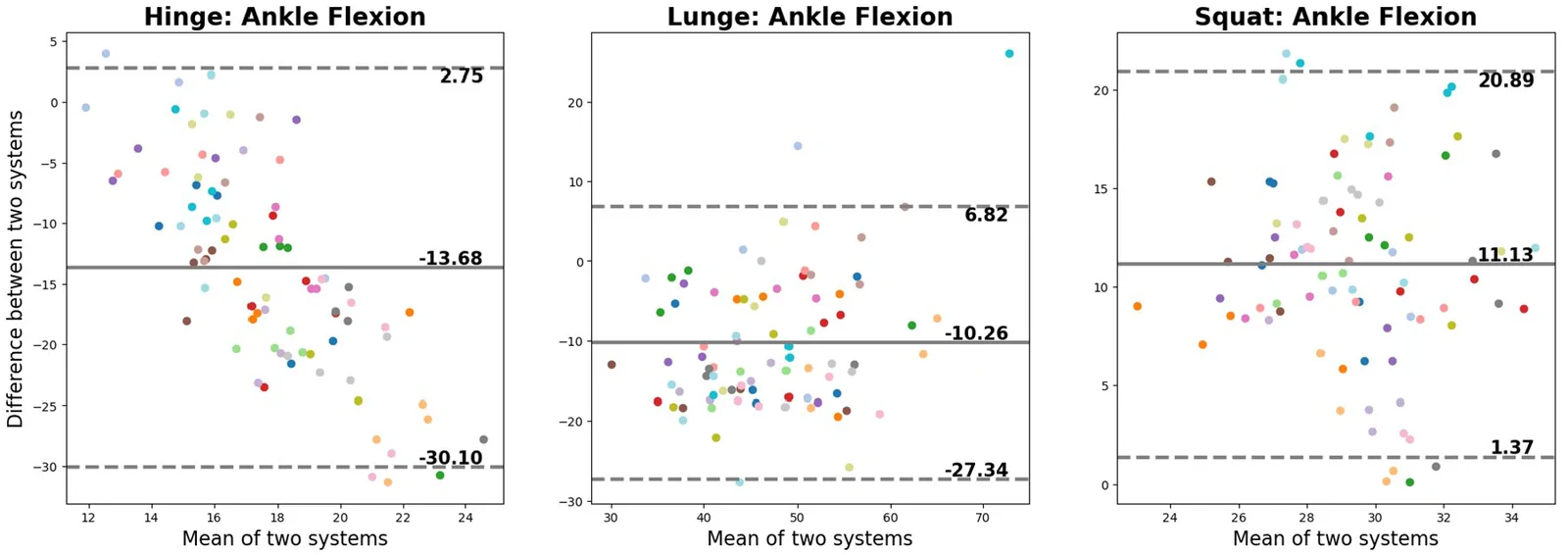
Bland–Altman plots of the peak ankle angle values measured by the markerless and marker-based motion capture systems. Differences were calculated as markerless measure − marker-based measure. Bias is the average of the differences between the two systems, and the limits of agreement are bias ±1.96 standard deviations of the differences.

Some linear relationships can be observed in the Bland-Altman plots. For hip internal rotation of both the lunge and the squat (Figure 3) and ankle flexion during the hinge (Figure 5), peak measurements from the markerless system tend to be greater than the marker-based system for smaller joint angles, and less than the marker-based system for larger joint angles. The opposite trend can be observed for the knee internal rotation angles during the hinge and squat, with the markerless system providing smaller estimates compared to the marker-based system for small joint angles, and greater measurements for larger joint angles. These linear relationships reflect a proportional bias, suggesting the differences between methods are not constant.

Two-way repeated measures ANOVAs revealed statistically significant differences between the bias values of the different tasks for hip flexion (F(2, 26) = 25.73), *p* < 0.001), hip adduction (F(2, 26) = 5.27), *p* = 0.01), hip internal rotation (F(2, 26) = 27.78), *p* < 0.001), knee flexion (F(2, 26) = 51.06, *p* < 0.001), ankle flexion (F(2, 26) = 116.90, *p* < 0.001), but not for knee varus (F(2, 26) = 1.96, *p* = 0.16) and knee internal rotation (F(2, 26) = 0.004), *p* > 0.99). Post hoc analyses were conducted for joint angles for which the repeated measures ANOVA was significant. For hip flexion, pairwise differences were revealed between the hinge and lunge (*p* < 0.001) and the lunge and squat (*p* < 0.001); the bias value of the lunge was smaller than the hinge and squat tasks. The post hoc analyses of hip adduction revealed differences between the hinge and squat tasks (*p* = 0.01), with the bias of the squat being greater. Hip internal rotation was significantly different between all pairwise comparisons of the three tasks (*p* < 0.05), with the hinge having the greatest bias and squat having the least. For knee flexion, bias values of the hinge were different from both the squat (*p* < 0.001) and the lunge (*p* < 0.001), where the bias of the hinge task was the greatest. Post hoc pairwise comparisons of ankle flexion revealed the bias values of the squat were different from those of both the hinge (*p* < 0.001) and the lunge (*p* < 0.001), with the squat demonstrating positive bias, whereas the hinge and lunge had negative bias values.

Repeated measure correlations of the peak joint angles measured by each system are listed in Table 4. The correlation coefficients of the peak measurements of hip flexion and ankle flexion demonstrate a low to moderate relationship between the two systems; the peak measurements of knee flexion demonstrate a moderate to strong relationship. Hip adduction, knee varus, and internal rotation of the hip and knee demonstrated low to very low correlation coefficients.

**Table 4.** Repeated measures correlations of the peak joint angle values measured by the markerless and marker-based motion capture systems.

Movement	Joint	Rotation	Correlation coefficient	p-value	95% Confidence Interval
Hinge	Hip	Flexion	0.31	0.01	0.09, 0.51
		Adduction	−0.23	0.06	−0.44, 0.01
		Internal rotation	−0.03	0.82	−0.26, 0.21
	Knee	Flexion	0.51	0.00	0.31, 0.66
		Varus	0.11	0.38	−0.13, 0.33
		Internal rotation	−0.08	0.55	−0.3, 0.16
	Ankle	Flexion	−0.15	0.20	−0.37, 0.08
Lunge	Hip	Flexion	0.57	0.00	0.39, 0.71
		Adduction	0.32	0.01	0.09, 0.51
		Internal rotation	0.15	0.21	−0.09, 0.37
	Knee	Flexion	0.69	0.00	0.55, 0.8
		Varus	−0.11	0.37	−0.33, 0.13
		Internal rotation	−0.10	0.42	−0.32, 0.14
	Ankle	Flexion	0.51	0.00	0.32, 0.67
Squat	Hip	Flexion	0.37	0.00	0.15, 0.56
		Adduction	−0.37	0.00	−0.56, −0.15
		Internal rotation	0.01	0.94	−0.22, 0.24
	Knee	Flexion	0.56	0.00	0.38, 0.7
		Varus	−0.07	0.55	−0.3, 0.16
		Internal rotation	0.36	0.00	0.13, 0.54
	Ankle	Flexion	−0.34	0.00	−0.53, −0.12

## Discussion

This study compared the lower limb joint angles of squat, hinge, and lunge tasks measured by a marker-based motion capture system and a markerless motion capture system. The gross motor patterns measured in situ demonstrated good agreement between the two systems in the sagittal plane; however, bias and RMSE values indicate systematic differences between the measurements of the two systems that may be affected by the assessed movement. Such differences may be attributed to differences in the available keypoints, number of cameras, filtering, and the approaches to computing segment position and orientation.

The discrepancy in both the number of markers available and the positions of these keypoints may contribute to the differences observed between the measurements of the two systems. With fewer keypoints available to the markerless system, the joint axes defined by the system and tracking of the local coordinate system are likely to be distinct from those of the marker-based system. Kadaba et al.^22^ conducted a sensitivity analysis of knee joint kinematics at different orientations of the flexion/extension axis during gait, revealing that while flexion/extension angles are relatively unaffected, the error of knee abduction/adduction and internal/external rotation angles were dependent on the flexion angle and magnitude of error of the defined axes. Given that the results of the present study demonstrated high peak cross-correlation values for hip and knee flexion (≥0.95), but low peak cross-correlation values for hip adduction (≤0.49), hip internal rotation (hinge = 0.46, lunge = 0.27), knee varus (≤0.43), and knee internal rotation (≤0.47), there is likely a discrepancy between the axes defined by the two systems. This poor correlation is further reflected in the proportional bias observed in the Bland–Altman plots for hip internal rotation during the lunge and squat, and knee internal rotation for the hinge and squat.

The chances of marker/landmark occlusion are reduced when a motion capture system utilizes more cameras. With the two-camera markerless system, estimations of unseen landmarks introduce error that may propagate to downstream calculations of three-dimensional joint kinematics.

The markerless system filtered joint angles using a 4th-order Butterworth filter, whereas joint angles from the marker-based system utilized a 6th-order Butterworth filter. While the rise time of a zero-lag Butterworth filter is not significantly affected by increasing the order of the filter, a higher-order filter will have greater attenuation of frequencies above the cutoff frequency.^23^ As such, it is possible that residual noise from the 4th-order filter and higher-frequency distortion by the 6th-order filter inflate the differences found between the measurements by the two systems.

The measurement of hip flexion was different between the two systems, based on the availability of markers/keypoints. The marker-based system calculated hip flexion as the angle between the pelvis and thigh segments; however, the markerless system did not have markers to model anterior-posterior pelvic tilt and instead calculated hip flexion as the angle between the trunk and thigh. This discrepancy in modeling may underlie the differences observed in hip flexion measurements between systems. An equivalent markerset for the marker-based system was not used because a comparison between the markerless system and the standard use case of the marker-based system was of interest. While high peak cross-correlation values (≥0.98) for all three tasks suggest agreement between the waveforms being measured, large RMSE values indicate systematic differences in the estimates. Further, better agreement of the bias and correlation coefficients of peak hip flexion was observed in the lunge task relative to the hinge and squat tasks. This better agreement may be due to the relatively limited spinal flexion occurring during the lunge, resulting in more similar orientations of the trunk and pelvis segments modeled by the different systems. Given the discrepancies in hip flexion measured between the two systems, the markerless system may not be the correct tool for measuring exact joint angles when spinal flexion is elicited by the movement under evaluation.

Different modeling approaches were used by the two systems. The marker-based system utilized a six DOF approach to estimating the pose and orientations of the segments, whereas the markerless system utilized an inverse kinematics approach. Based on the different joint constraints and joint center definitions used to calculate joint angles, the kinematic outputs of the two different systems will be non-equivalent. This non-equivalence would likely explain the negative correlations of peak ankle angle for the hinge and squat tasks, as the “zero” position of the ankle joint defined in each of the models used to calculate joint angles is different. A previous comparison of IMUs and an optoelectronic system for whole-body motion analysis found that the distinct biomechanical models were the greatest contributor to the differences between the two systems.^24^ While adapting the markerset and modeling of the marker-based system to match those of the markerless system would reduce the differences between the two systems, the current study presents a comparison between the two systems as they would be utilized under normal circumstances.

Discrepancies between the kinematic outputs of marker-based and markerless motion capture systems are to be expected, given the potential sources of error affecting each system. Marker misplacement and soft tissue artifact have been identified as the two greatest sources of error in marker-based motion capture.^3,4^ The rotational axes of a joint are defined by anatomical landmarks; as such, marker placement and the subsequent propagation of orientation error affects the joint angles measured.^25^ Soft tissue artifact is the relative motion between a skin-affixed marker and the underlying bony landmark; the pattern of error is both task and subject dependent.^3^

The kinematic outputs of a markerless motion capture system are influenced by the quality of the training data used to train the pose estimation model. Training datasets are manually labeled, resulting in inherent subjectivity. Further, the variety of images in the training set can bias or result in weaknesses in the algorithm’s ability to detect people and keypoints.^26^ Additionally, the keypoints identified by the computer vision model affect the inverse kinematics to calculate joint positions. These factors may influence the high bias and RMSE values observed in the present investigation, suggesting that the angles measured by the markerless system may not be accurate.

The lower limb gait kinematics of other markerless motion capture systems have been evaluated against marker-based systems.^5,11^ An eight-camera commercial markerless motion capture system captured similar patterns and ranges of motion for flexion/extension of the hip, knee, and ankle, and abduction/adduction of the hip and knee; the greatest average RMSE (13.2°) occurred in knee internal/external rotation.^5^ Similarly, an open-sourced two-smartphone based system reported agreement in hip, knee, ankle flexion/extension, and hip abductiom/adduction, with the greatest average RMSE (6.6°) occurring in hip internal/external rotation.^11^ These systems reported lower RMSE values than the ones calculated in the present investigation; however, it should be noted that that unlike the previous investigations, the tasks evaluated in the current evaluation were not gait. The RMSE values observed in this study are likely inflated by the differences in markersets, digital filtering, and kinematic modeling used by the two systems.

Given the inherent differences between systems and the subsequent processing performed, the findings of the present study should be interpreted with caution. The general agreement of the flexion angle waveforms between the two systems indicates the markerless system can capture the coordination and pattern of the evaluated movement, despite outputting non-equivalent joint angles. The 3D markerless motion capture system would be a useful tool for identifying gross coordination strategies in the sagittal plane; however, marker-based systems would be more appropriate for the exact joint angle measurements.

### Limitations

Time series data of the systems were aligned using the peak left hip flexion value for each trial, rather than visual^6^ or force-based^5^ events to mark the beginning and end of each trial. While the cross-correlation values of hip flexion were high for all three movement tasks, discrepancies in the relative timing of the peak left hip flexion values between systems may lead to inflated difference values calculated downstream due to the misalignment of the corresponding signals from the two systems. Synchronization of the signals of the two waveforms using events independent of the measured signals would not only ensure proper alignment but also allow comparison of time-normalized movement tasks.

The markerless system used relatively fewer keypoints to conduct pose estimation, compared to the marker-based system. Because the markerless system did not have keypoints to define anterior-posterior pelvic tilt, this system calculated hip flexion as the relative angle between the thigh and the trunk, rather than the thigh and pelvis in the marker-based system. Additionally, joint angles in the markerless system were calculated using the inverse kinematics approach, whereas the marker-based system used a six DOF approach. These differences in methods between the two systems can be attributed to some of the differences observed between the calculated joint angles.

The markerless setup for the present study utilized the cameras from two iOS devices, which is the minimum number of cameras required. This setup was done to establish the baseline performance of the system. Synchronizing additional cameras to the system would be expected to improve the accuracy of the kinematic estimates by reducing the likelihood of landmark occlusion.

Data were collected at 60 Hz by the markerless system, given the relatively slow motion of the evaluated movements. For faster movements, this system can collect data at 120 Hz. Based on the Nyquist-Shannon sampling theorem, for movements containing frequencies of 60 Hz or higher, cameras permitting a sampling frequency greater than 120 Hz would be required.

The present study was conducted in a biomechanics research laboratory with minimal background occlusion. In the future, testing in less controlled, ecologically valid settings will be important for in-the-wild applications, where the markerless system is intended to be used.

## Conclusion

The present study compared the lower limb kinematics measured by a novel markerless motion capture system against a gold standard marker-based system. Flexion at the knees and hips demonstrated strong agreement between patterns measured by the two systems, despite offsets between the systems. The agreement of the measurements of hip internal rotation and ankle flexion were dependent on the task being performed. Measurements of hip adduction, knee internal rotation, and knee varus demonstrated poor agreement between systems for the three analyzed tasks. The markerless motion capture system demonstrated potential as an out-of-the-box, lower-cost, easy-to-use solution for evaluating human movement. Further refinement of the computer vision and biomechanical models is needed to improve the validity of the kinematic measurements.

## Supplemental Material

sj-jpg-1-pip-10.1177_17543371251348697 – Supplemental material for A comparison of lower extremity squat, lunge, and hip hinge kinematics between marker based and markerless motion capture systems Supplemental material, sj-jpg-1-pip-10.1177_17543371251348697 for A comparison of lower extremity squat, lunge, and hip hinge kinematics between marker based and markerless motion capture systems by Kristie Liu, Steven Hirsch, Pratham Singh, Timothy CY Chan, Timothy A Burkhart and Michael G Hutchison in Proceedings of the Institution of Mechanical Engineers, Part P: Journal of Sports Engineering and Technology

sj-jpg-2-pip-10.1177_17543371251348697 – Supplemental material for A comparison of lower extremity squat, lunge, and hip hinge kinematics between marker based and markerless motion capture systems Supplemental material, sj-jpg-2-pip-10.1177_17543371251348697 for A comparison of lower extremity squat, lunge, and hip hinge kinematics between marker based and markerless motion capture systems by Kristie Liu, Steven Hirsch, Pratham Singh, Timothy CY Chan, Timothy A Burkhart and Michael G Hutchison in Proceedings of the Institution of Mechanical Engineers, Part P: Journal of Sports Engineering and Technology

sj-jpg-3-pip-10.1177_17543371251348697 – Supplemental material for A comparison of lower extremity squat, lunge, and hip hinge kinematics between marker based and markerless motion capture systems Supplemental material, sj-jpg-3-pip-10.1177_17543371251348697 for A comparison of lower extremity squat, lunge, and hip hinge kinematics between marker based and markerless motion capture systems by Kristie Liu, Steven Hirsch, Pratham Singh, Timothy CY Chan, Timothy A Burkhart and Michael G Hutchison in Proceedings of the Institution of Mechanical Engineers, Part P: Journal of Sports Engineering and Technology

## Data Availability

Data available on request from the authors.
